# Recurrent/moderate hypoglycemia induces hippocampal dendritic injury, microglial activation, and cognitive impairment in diabetic rats

**DOI:** 10.1186/1742-2094-9-182

**Published:** 2012-07-25

**Authors:** Seok Joon Won, Byung Hoon Yoo, Tiina M Kauppinen, Bo Young Choi, Jin Hee Kim, Bong Geom Jang, Min Woo Lee, Min Sohn, Jialing Liu, Raymond A Swanson, Sang Won Suh

**Affiliations:** 1 Department of Neurology, University of California at San Francisco and Veterans Affairs Medical Center, San Francisco, CA, USA; 2 Department of Neurosurgery, University of California San Francisco and Veterans Affairs Medical Center, San Francisco, CA, USA; 3 Department of Anesthesiology, Inje Paik Hospital, Inje University, School of Medicine, Seoul, Korea; 4 Inha University, Department of Nursing, Incheon, Korea; 5 Department of Physiology, Hallym University, College of Medicine, Chuncheon, 200-702, Korea

**Keywords:** Recurrent/moderate hypoglycemia, Dendrite, Superoxide, Lipid peroxidation, Neuron death, Microglial activation, NADPH oxidase, Apocynin

## Abstract

**Background:**

Recurrent/moderate (R/M) hypoglycemia is common in type 1 diabetes. Although mild or moderate hypoglycemia is not life-threatening, if recurrent, it may cause cognitive impairment. In the present study, we sought to determine whether R/M hypoglycemia leads to neuronal death, dendritic injury, or cognitive impairment.

**Methods:**

The experiments were conducted in normal and in diabetic rats. Rats were subjected to moderate hypoglycemia by insulin without anesthesia. Oxidative stress was evaluated by 4-Hydroxy-2-nonenal immunostaining and neuronal death was determined by Fluoro-Jade B staining 7 days after R/M hypoglycemia. To test whether oxidative injury caused by NADPH oxidase activation, an NADPH oxidase inhibitor, apocynin, was used. Cognitive function was assessed by Barnes maze and open field tests at 6 weeks after R/M hypoglycemia.

**Results:**

The present study found that oxidative injury was detected in the dendritic area of the hippocampus after R/M hypoglycemia. Sparse neuronal death was found in the cortex, but no neuronal death was detected in the hippocampus. Significant cognitive impairment and thinning of the CA1 dendritic region was detected 6 weeks after hypoglycemia. Oxidative injury, cognitive impairment, and hippocampal thinning after R/M hypoglycemia were more severe in diabetic rats than in non-diabetic rats. Oxidative damage in the hippocampal CA1 dendritic area and microglial activation were reduced by the NADPH oxidase inhibitor, apocynin.

**Conclusion:**

The present study suggests that oxidative injury of the hippocampal CA1 dendritic region by R/M hypoglycemia is associated with chronic cognitive impairment in diabetic patients. The present study further suggests that NADPH oxidase inhibition may prevent R/M hypoglycemia-induced hippocampal dendritic injury.

## Introduction

Recurrent/moderate (R/M) hypoglycemia, which is more common than severe hypoglycemia in type 1 diabetes patients (frequency is 0.1 to 0.3 episode/patient per day), is usually corrected by patients themselves or unrealized. Therefore, these episodes may be easily forgotten and remain untreated [1-4]. Although moderate hypoglycemia is not life-threatening, if recurrent, it may have serious clinical implications. Poor academic performance in diabetic children and memory impairment in adults with diabetes are viewed as areas of increasing public concern. These problems are typically thought to result from hypoglycemic attacks, which occur more frequently during intensive insulin therapy [5-8].

The normal range for human blood glucose concentration is 3.9 to 7.1 mM (1 mM = approximately 18 mg/dl), and hypoglycemia is broadly defined as glucose concentrations that fall below this range. Reducing food intake does not reduce blood glucose levels below 2.8 mM, even with prolonged fasting [9], however, reductions below 1 mM can be induced by the administration of insulin or drugs such as sulfonylurea derivatives. Hypoglycemic brain injury occurs most frequently in diabetic patients attempting tight glucose control [10,11], and consequently the risk of hypoglycemic brain injury is the major factor limiting tight glucose control. Although there is no strict definition, hypoglycemia is generally classified as mild (3.2 to 3.6 mM), moderate (2.3 to 3.2 mM) and severe (<2 mM) hypoglycemia in humans [12]. While most diabetic patients experience mild to severe hypoglycemia at times, very few experience severe hypoglycemia to a degree that results in coma.

Studies using mice and rats indicate that widespread neuronal death does not occur unless blood glucose concentration falls below 1 mM and cortical electroencephalographic (EEG) activity remains isoelectric (silent) for at least 10 min. However, it is still possible to detect scattered neuronal death, even at early stages of electro-cerebral silence or sustained blood glucose concentrations just in excess of 1 mM [13-16]. Case studies suggest that a similar degree of hypoglycemia is required to induce brain injury in humans, although this may vary in children, susceptible individuals with repeated hypoglycemia and the presence of co-morbid factors [17-22].

The main risk factors for cognitive impairment in diabetes are considered to be age, duration of diabetes, and coexistent microvascular and macrovascular complications [23]. Diabetes should be treated as a risk factor for cognitive impairment, and likewise, cognitive impairment is associated with poorer ability in diabetes self-care and decreased adherence to anti-diabetic treatment, setting the stage for a potential vicious cycle of decline in quality of life and disease management.

Children with type 1 diabetes who suffer recurrent and severe hypoglycemia while younger than 5 years old have impaired mental abilities later in life [6,24]. The combination of an early onset of diabetes and recurrent episodes of hypoglycemia appears to be associated with reduced attention and spatial memory in adolescence [24-27]. Within the diabetic group, verbal intelligence was reduced with increased exposure to hyperglycemia, but not to hypoglycemia. In contrast, spatial intelligence and delayed recall were reduced only with repeated hypoglycemia, particularly when hypoglycemic episodes occurred before the age of 5 years [28].

Recurrent episodes of mild/moderate hypoglycemia are associated with a decreased perception of the hypoglycemic state and blunted secretion of counter-regulatory hormones, phenomena termed ‘hypoglycemia unawareness’ and ‘hypoglycemia-associated autonomic failure’ (HAAF), respectively [29-31]. In diabetic patients, even mild to moderate hypoglycemia may produce a significant increase in low-frequency EEG activity [32] and impair cognitive function [33]. Several studies have sought to determine the cognitive impact of recurrent hypoglycemia, but results from clinical studies have been mixed and the question of whether recurrent exposure to severe hypoglycemia promotes long-term cognitive dysfunction is unresolved.

There have been several studies performed to determine whether neuronal death is induced after moderate hypoglycemia, which is defined as low blood glucose levels (below 2 mM blood glucose for more than 2 h) without the presence of isoelectric EEG (iso-EEG). These studies concluded that moderate hypoglycemia induced scattered neuronal death in the cerebral cortex layer 2–3 [34], but not in the hippocampus. Yamada *et al*. also found that moderate hypoglycemia led to no hippocampal neuronal death, however, they did observe significantly deteriorated synaptic plasticity, demonstrated by an inability to induce long term potentiation (LTP) at CA1 synapses [35]. Thus, we speculated that repetitive episodes of moderate hypoglycemia might lead to synaptic injury in the hippocampus in the absence of apparent neuronal somatic injuries in the hippocampus, and consequently, the development of cognitive impairments.

To date, several hypoglycemia experiments have been performed with normal (non-diabetic) adult rodents; therefore the clinical implication of these studies is not readily apparent since moderate hypoglycemia commonly occurs in juvenile type 1 diabetes patients. Therefore, the present study was conducted in 1-month-old young rats that were rendered diabetic by streptozotocin (STZ) injection, mimicking juvenile type 1 diabetes.

To test our hypothesis, the present study has addressed five questions. First, does a single episode of moderate hypoglycemia (without anesthesia and without iso-EEG) cause dendritic oxidative injury in the hippocampus? Second, if not, do repeated episodes of moderate hypoglycemia cause dendritic oxidative injury in the hippocampus? If so, third, do repeated episodes of moderate hypoglycemia cause cognitive impairment at a later time point? Fourth, is there a difference in cognitive function between non-diabetic and diabetic rats, with respect to the synaptic effects of R/M hypoglycemia? Fifth, is oxidative injury or microglial activation after R/M hypoglycemia mediated by NADPH oxidase?

## Materials and methods

The surgical and animal care procedures were in accordance with the guidelines of the Institutional Animal Care and Use Committee of the San Francisco Veterans Affairs Medical Center (animal welfare assurance number A3476-01) and of the Hallym University (Hallym 2011–44). This manuscript was written up in accordance with the ARRIVE (Animal Research: Reporting In Vivo Experiments) guidelines [36].

### Type 1 diabetes rat

For the type 1 diabetes model, rats received repeated intraperitoneal injections of streptozotocin (STZ). One-month-old male Sprague Dawley rats were injected with 50 mg/kg STZ (twice; second injection occurred 24 h after the first injection, i.p.). Rats displaying fasting blood glucose levels higher than 200 mg/dl 7 days after the first injection were used in this experiment. All rats injected with STZ showed typical symptoms of diabetes such as polydipsia, polyphagia, and polyuria. Although the STZ-induced diabetes mellitus (DM) model has certain caveats, this model offers a very effective technique that can be used in most rodents [37]. Fasting blood glucose levels in STZ-treated rats were left uncorrected and fell in the range of 10 to 20 mM.

### R/M hypoglycemia

One week after STZ injection, R/M hypoglycemia was induced without anesthesia. After overnight fasting, rats were given an insulin injection (5 U/kg, i.p.) to induce moderate hypoglycemia, as previously described with minor modification [38]. After insulin injection, blood glucose was measured from the tail vein using ACCU-CHEK glucometer (Roche, Indianapolis, IN, USA) at 30-min intervals. Blood glucose levels between 1 and 2 mM were maintained for 1 h. Blood glucose level was increased by intraperitoneal injection of glucose (25%/1 mL/bolus) 1 h after moderate hypoglycemia (Table 1). In the present study we defined ‘moderate hypoglycemia’ as sustained (1 h) blood glucose concentrations between 1 to 2 mM without coma. This process was repeated for 5 consecutive days (Additional file 1: Figure S1). Rats were randomly assigned to two groups; one for studying the role of oxidative stress and the other group for cognitive testing 6 weeks post-insult. To test whether NADPH oxidase activation is involved in R/M hypoglycemia-induced oxidative injury in hippocampal dendrites, the NADPH oxidase assembly inhibitor, apocynin, was injected intraperitoneally (15 mg/kg, Sigma, St Louis, MO, USA) immediately after R/M hypoglycemia. Since half-life of apocynin is about 1 h [39], we believe that apocynin may inhibit glucose reperfusion-induced NADPH oxidase activation for 2 to 3 h. Apocynin was dissolved in 1% DMSO (dimethylsulphoxide) and control rats for these studies received equal volumes of vehicle alone. This process was repeated for 5 consecutive days (Additional file 1: Figure S1).

**Table 1:** **Change in mean arterial blood glucose levels before insulin injection, during moderate hypoglycemia and after glucose reperfusion in diabetic and non-diabetic rats (mM** ± **s.e.m.)**

	**Before insulin injection**	**During moderate hypoglycemia**	**After glucose reperfusion**
Non-diabetic rats (*n* = 7)	4.72 ± 0.27	1.46 ± 0.04	5.61 ± 0.25
Diabetic rats (*n* = 7)	16.43 ± 0.81	1.48 ± 0.10	17.31 ± 0.77

### Acute/severe (A/S) hypoglycemia

As a means of comparison with R/M hypoglycemia, A/S hypoglycemia with diabetic rats was also performed in the present study. Insulin-induced A/S hypoglycemia was induced in rats as previous described, with the period of severe hypoglycemia defined here as the interval during which the EEG exhibited an isoelectric state for 30 min [13,15]. Severe hypoglycemia was induced by intraperitoneal injection of 30 U/kg of regular insulin (Novolin-R, Novo Nordisk, Clayton, NC, USA) (Additional file 2: Figure S2).

### Evaluation of neuron degeneration

Neuronal death after R/M or A/S hypoglycemia was evaluated after a 7-day survival period. Rats were intracardially perfused with 0.9% saline followed by 4% paraformaldehyde (PFA). The brains were post-fixed with 4% PFA for 24 h and then incubated with 20% sucrose for cryoprotection. For H&E staining, coronal 25 μm sections were conventionally stained [40]. Brain sections were also prepared for the Fluoro-Jade B staining (FJB) [15,41]. Degenerating cells were detected with 450 to 490 nm excitation and a 515 nm emission filter. Five coronal sections were collected from each animal by starting 4.0 mm posterior to Bregma, and collecting every third section until five sections were in hand. These sections were then coded and given to a blinded experimenter who counted the number of degenerating neurons in the hippocampal CA1.

### Evaluation of oxidative stress

Oxidative injury was estimated by evaluating levels of the lipid peroxidation product, 4HNE (4-hydroxy-2-nonenal). Immunostaining with microtubule-associated protein 2 (MAP2; Millipore Co, Billerica, MA, USA) and 4HNE (Alpha Diagnostic Intl. Inc., San Antonio, TX, USA) antibodies was performed as described previously [42]. Three sections were analyzed from each brain, taken at 80 μm intervals to span the hippocampus. 4HNE signal intensity was expressed as the ratio of the mean fluorescence in SR of hippocampal CA1 to fluorescence in the lateral ventricles.

### Immunohistochemistry

For evaluation of dendritic damage, the sections were immunostained with a rabbit antibody to rat MAP2 (Millipore Co, Billerica, MA, USA) at a 1:250 dilution. For evaluation of microglial activation, the sections were immunostained with a mouse antibody to rat CD11b (Serotec, Raleigh, NC, USA) at a 1:200 dilution. Microglia activation was evaluated by an observer blinded to experimental conditions. Three sections from each animal were evaluated for scoring. Microglia activation criteria were depending on number of CD11b immunoreactive cells and their morphology as published previously [43].

### Behavioral assessments

Rats were handled for 5 min each per day and acclimated in the behavioral test room for 1 week prior to the start of behavior testing. Six weeks following the sham hypoglycemia or R/M hypoglycemia, rats were first tested in the novel open field test to evaluate the effects of diabetes and recurrent hypoglycemia on locomotor activity and exploratory behavior. In the open field test, rats were placed in a brightly lit, square Plexiglas enclosure (40 x 40 inches), surrounded by automated infrared photocells interfaced with a computer (Hamilton & Kinder, San Diego, CA, USA) to record the data. On each of 3 consecutive days, open field activity was recorded for 10 min after an initial 1-min adaptation period. For analysis of the exploratory behavior, the arena was divided on a zone map consisting of a center region (15 × 15 inches), four corner regions of 7.5 × 7.5 inches each, and a peripheral region (the remaining area).

Subjects then underwent testing in the Barnes maze test to evaluate spatial learning and memory [44]. Rats from each treatment group were randomly assigned to locate the escape tunnel from one of the three predetermined locations to rule out spatial preference. Moderately noxious stimuli, blowing fans and 500 LUX of bright light, were used to increase the incentive in finding the escape tunnel. Noldus EthoVision video tracking system (Noldus, Leesburg, VA, USA) was used to record and analyze the data. Rats were trained to locate the escape tunnel in two successive daily sessions for 5 days (three trials per session, 3 min per trial) with a 1-h intersession interval from different counterbalanced starting positions.

### Statistical analysis

Data are expressed as means ± s.e.m. and assessed by one-way ANOVA followed by either the Tukey-Kramer test for multiple comparisons between groups or the Dunnett’s test for comparisons of multiple groups against a control group with Statview 5.0.1 software (SAS Institute Inc., Cary, NC, USA). Behavioral data were compared using two-way repeated measure ANOVA (RANOVAs) followed by *post hoc* pairwise comparisons using the Bonferroni test when appropriate. Values of *P* < 0.05 were considered as significant.

## Results

### A/S hypoglycemia induces cortical and hippocampal neuronal death in diabetic rats

**Figure 1:**
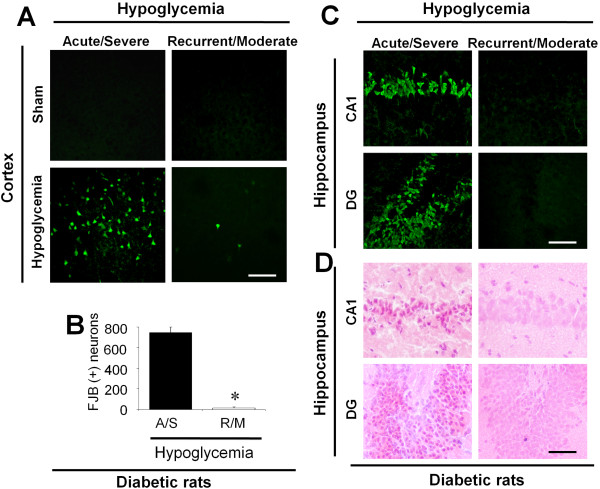
**Neuronal death in the cerebral cortex and hippocampus after severe or moderate hypoglycemia in diabetic rats.** (**A**) In sham hypoglycemia-operated rats, neuronal death (FJB (+) neurons) was not detected in diabetic rats. In both diabetic and non-diabetic rats, recurrent/moderate (R/M) hypoglycemia shows only occasional neuronal death in the cerebral cortex while acute/severe (A/S) hypoglycemia shows widespread neuronal death in the superficial layer of cortex at 7 days after hypoglycemia. Bar = 100 μm. (**B**) Bar graph represents counted degenerating neurons in the cerebral cortex. Data are mean + s.e.m.; *n* = 5 in R/M hypoglycemia, *n* = 7 in A/S hypoglycemia * *P* < 0.05. (**C**) R/M hypoglycemia shows no neuronal death (FJB (+) neurons) in the hippocampal CA1 and DG cell layer while severe hypoglycemia shows substantial neuron death in the hippocampus at seven days after hypoglycemia. Bar = 100 μm. (**D**) Light microscopic images represent hematoxylin & eosin (H&E) stained hippocampus from CA1 and DG cell layer. R/M hypoglycemia shows no neuronal death (eosinophilic) in the hippocampal CA1 and DG cell layer while severe hypoglycemia shows substantial neuronal death in the hippocampus at seven days after hypoglycemia. Bar = 100 μm.

Before initiating the R/M hypoglycemia experiments, diabetic rats were subjected to A/S hypoglycemia to test whether STZ-injected diabetic rats also have similar patterns of neuronal injury as seen in previous studies using non-diabetic rats (Figure 1). We found that, in the cerebral cortex, A/S hypoglycemia produced about twice as much neuron death in diabetic rats as in non-diabetic rats, compared to other historical controls [13,45] and our previous study [15,42]. However, in the hippocampus, diabetic and non-diabetic rats showed a similar degree of neuronal death after A/S hypoglycemia. This result is very similar to that reported by Bree *et al*. [46].

### R/M hypoglycemia produces infrequent, sparse neuronal death in the cerebral cortex

Neuronal death was evaluated in the cerebral cortex of STZ-induced diabetic rats after R/M hypoglycemia (Figure 1A and B). Neuronal injury evaluated by FJB staining at 7 days after R/M hypoglycemia showed infrequent, sparse neuronal death in the parietal region of cerebral cortex. Only three out of five diabetic rats exhibited FJB(+) cells in the cerebral cortex.

### R/M hypoglycemia does not produce neuronal death in the hippocampus

Neuronal injury evaluated by H&E or FJB staining at 7 days after A/S hypoglycemia showed widespread neuronal death in the hippocampus of type-1 diabetes mellitus model rats. However, R/M hypoglycemia produced no hippocampal neuronal death in the diabetic rats (Figure 1C and D).

### A single episode of moderate hypoglycemia does not induce oxidative injury in hippocampal CA1 dendrites

**Figure 2:**
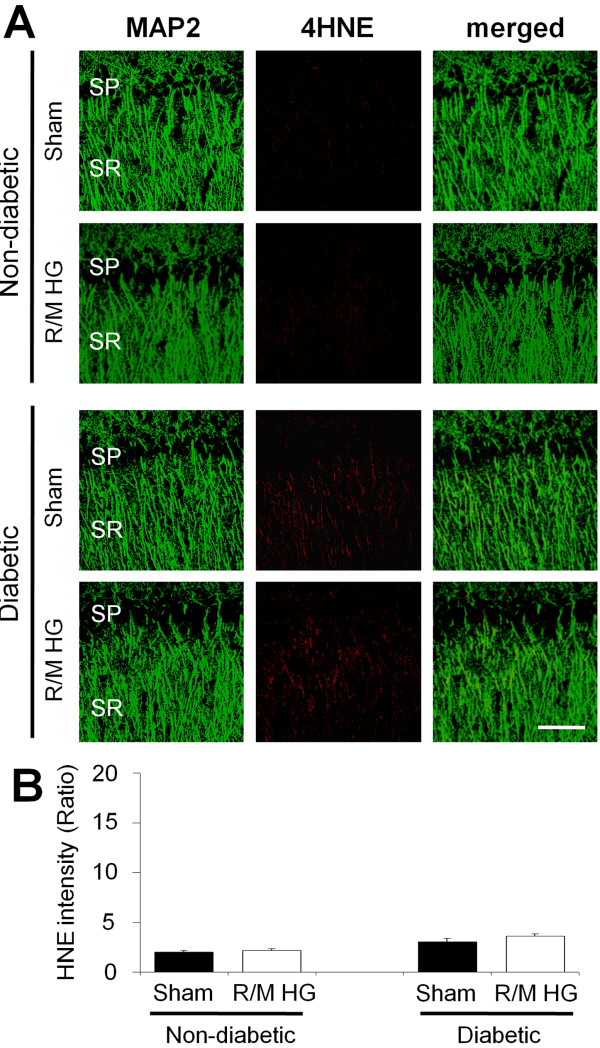
**A single episode of moderate hypoglycemia does not induce oxidative injury in the hippocampal CA1 dendritic area.** (**A**) Fluorescent images show MAP2 (green) and 4HNE (red) antibody stained stratum radiatum (SR) of hippocampal CA1 area. The 4HNE intensity in the dendritic area after a single episode of moderate hypoglycemia (M-HG) is almost identical between non-diabetic (saline treated) and diabetic rats. SP, Stratum pyramidale. Bar = 100 μm. (**B**) Bar graph represents quantitated 4HNE fluorescent intensity from the SR area of hippocampal CA1 area. Data are mean + s.e.m.; *n* = 5.

To test whether a single episode of moderate hypoglycemia induces oxidative injury at or proximal to hippocampal dendrites, rat hippocampus was evaluated at 24 h after a single episode of moderate hypoglycemia. Here we found only low levels of 4HNE fluorescent signal in SR area of hippocampus after a single episode of moderate hypoglycemia in either the non-diabetic or the diabetic rats (Figure 2).

### R/M hypoglycemia induces significant oxidative injury in the hippocampal stratum radiatum area

**Figure 3:**
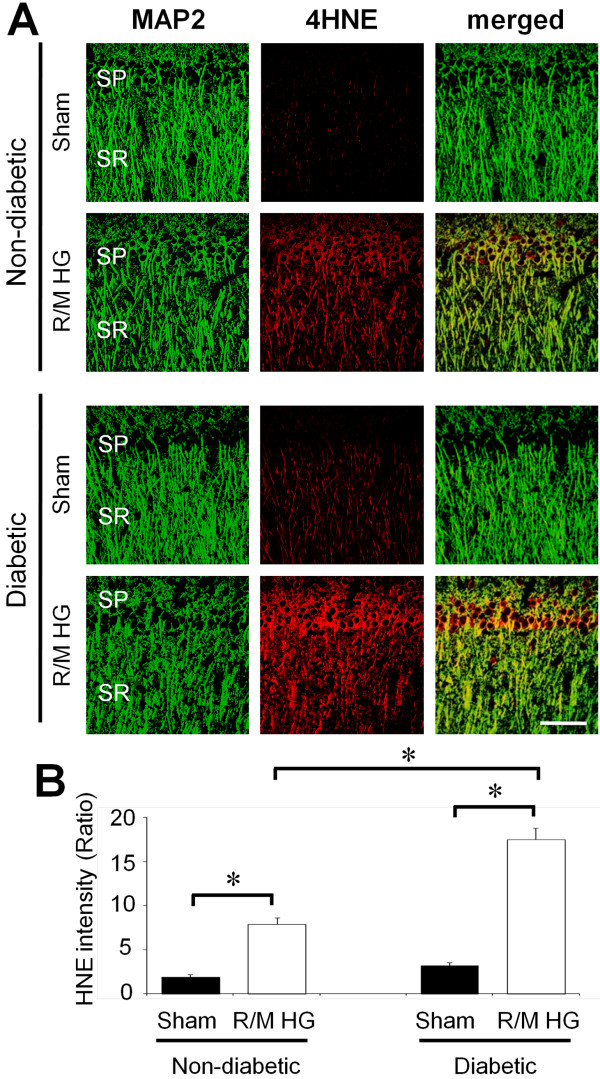
**Repeated episodes of moderate hypoglycemia induce a significant increase in oxidative injury in the hippocampal CA1 dendritic area.** (**A**) Fluorescent images show MAP2 (green) and 4HNE (red) antibody stained stratum radiatum (SR) of hippocampal CA1 area. The 4HNE intensity in the dendritic area after R/M hypoglycemia (R/M HG) is substantially increased. This 4HNE intensity increase is much higher in the diabetic rats, which suggests oxidative injury in the dendritic area of hippocampus is worse in the diabetic animal. SP, Stratum pyramidalae. Bar = 100 μm. (**B**) Bar graph represents quantitated 4HNE fluorescent intensity from the SR area of hippocampal CA1 area. Data are mean + s.e.m.; *n* = 7, * *P* < 0.05.

Since a single episode of moderate hypoglycemia induced no obvious oxidative injuries in the SR, rats were exposed to recurrent hypoglycemia for 5 consecutive days. One day after the final episode of R/M hypoglycemia (after five episodes of R/M hypoglycemia), brains were examined to determine if oxidative injury had occurred in hippocampal dendritic area. Compared with a single episode of moderate hypoglycemia, exposure to five consecutive episodes leads to significant oxidative injury in the hippocampal dendrite area. 4HNE fluorescent intensity was increased by 419% in the SR area of hippocampus of non-diabetic rats. 4HNE fluorescence intensity was increased by 549% in the diabetic rats over non-diabetic rats (Figure 3).

### R/M hypoglycemia induces microglial activation

**Figure 4:**
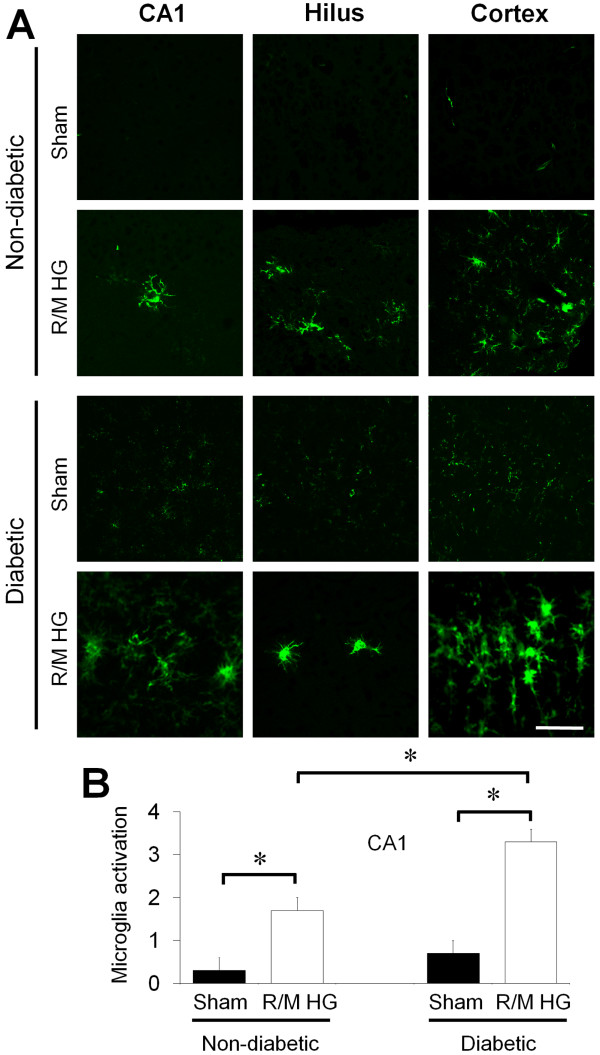
**R/M hypoglycemia increases microglial activation in the hippocampus and in the cortex.** (**A**) Brain sections were harvested at one day after the last episode of moderate hypoglycemia and immunostained with CD11b. R/M hypoglycemia-induced microglial activation in the hippocampus and in the cerebral cortex is apparent in both non-diabetic (Saline) and diabetic (STZ) rats. Diabetic rats show substantially increased microglial activation in the hippocampal CA1, hilus, and cortex compared to non-diabetic rats. Scale bar = 100 μm. (**B**) Quantification of microglial activation was performed from hippocampal CA1 pyramidal area. Microglial activation is quantified based on morphological change and intensity of CD11b immunostaining. As shown in the images, microglial activation is aggravated in diabetic rats. Data are mean + s.e.m.; *n* = 5, * *P* < 0.05.

We tested whether R/M hypoglycemia also induces microglial activation in the cerebral cortex and the hippocampus. Rats were exposed for 5 days to R/M hypoglycemia and brains were harvested after the final episode of R/M hypoglycemia. Compared to sham hypoglycemia operated rats, microglia were activated in the cerebral cortex and in the hippocampus after R/M hypoglycemia in normoglycemic rats. The degree of microglial activation in the hippocampus of diabetic rats was significantly higher than in normoglycemic rats (>194%, Figure 4).

### R/M hypoglycemia worsens impairment in spatial learning and memory in diabetic rats

**Figure 5:**
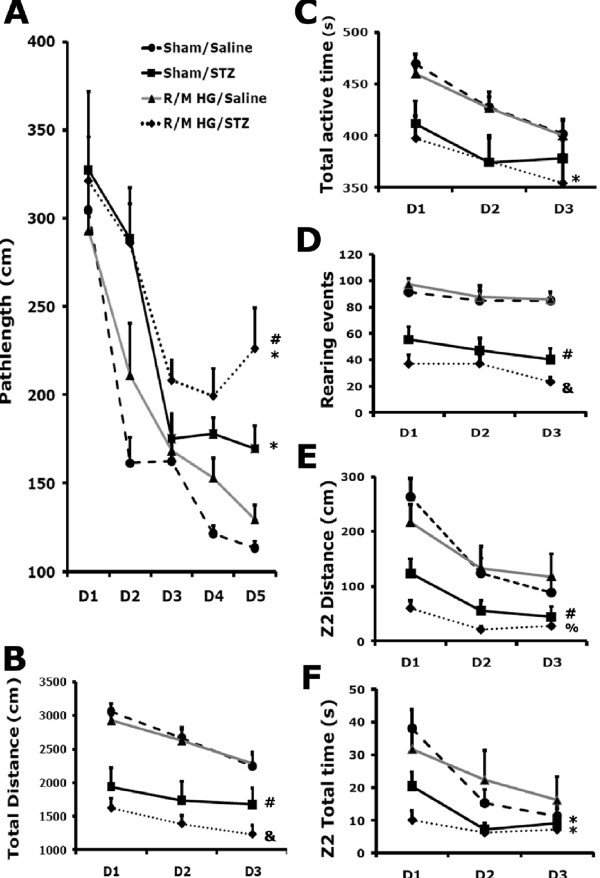
**R/M hypoglycemia induces cognitive impairment in diabetic rats.** STZ induced impairment in spatial learning and memory by increasing the path length traveled in the BM test (*: sham/saline *vs*. sham/STZ: *P* < 0.05; #: R/M-HG/saline *vs*. R/M-HG/STZ: *P* < 0.01). R/M hypoglycemia (R/M-HG) induced spatial learning and memory impairment only in the diabetic (*: R/M-HG/STZ *vs*. sham/STZ: *P* < 0.05), but not in non-diabetic rats (R/M-HG/saline *vs.* sham/saline: *P* = 0.65) (**A**). Diabetes but not R/M-HG reduced overall locomotor activity as evidenced by reduced total distance traveled (**B**), total active time (**C**) and vertical activity in rearing (**D**). Similarly, diabetes but not R/M-HG altered exploratory behavior by reducing activity in the center zone (Z2) of an open field (**E, F**) (D1, D2, and D3 = Day1, Day2, and Day3). *Post-hoc* analysis suggests that there are significant differences between R/M-HG/saline and R/M-HG/STZ in total distance (labeled as & in B), total active time (labeled as * in C), rearing events (labeled as & in D), Z2 distance (labeled as % in E) and Z2 total time (labeled as * in F). There were also significant differences between sham/saline and sham/STZ in total distance (# in B), rearing events (& in D), Z2 distance (# in E) and Z2 total time (* in F). *: P < 0.05, #: *P* < 0.01, %: *P* < 0.005, and &: *P* < 0.001.

We next examined whether hippocampal oxidative injury observed following R/M hypoglycemia correlated with the presence of memory deficits. Rats were subjected to testing designed to evaluate spatial learning in the Barnes maze that heavily relies on hippocampal function. Over the 5 days of training, all groups learned the spatial task as evidenced by a progressive reduction in the distance traveled to reach the escape tunnel in the Barnes maze test (F_4, 264_ = 95.7; *P* < 0.01). There was also a significant difference in the performance during the acquisition phase of the Barnes maze test between groups. In general, diabetic rats traveled significantly longer distances to reach the escape tunnel compared to non-diabetic rats (Two-way RANOVA, STZ effect: F_1, 66_ = 17.5; *P* < 0.01) (Figure 5A). *Post-hoc* analysis suggests that diabetic rats with R/M hypoglycemia performed significantly worse than either diabetic sham hypoglycemic rats (*P* < 0.05), or non-diabetic rats with R/M hypoglycemia (*P* < 0.01) (Figure 5A).

### Diabetes, but not R/M hypoglycemia, alters exploratory behavior

To investigate whether R/M hypoglycemia induces activity changes in either non-diabetic or diabetic animals, rats were subjected to an open field test at 6 weeks after the final episode of R/M hypoglycemia. All groups were able to habituate over 3 days of testing in the novel open field (Figure 5B to D, total path length: *P* < 0.01; total active time: *P* < 0.0001; rearing: *P* < 0.01). Diabetes had a strong effect on locomotion (Figure 5B to D, total path length: *P* < 0.01; total active time: *P* < 0.01; rearing: *P* < 0.01), while R/M hypoglycemia did not reduce locomotion (5B to D, total path length: *P* = 0.16; total active time: *P* = 0.49; rearing: *P* = 0.40). Because the novel environment in the open field concurrently evokes both anxiety and exploration [47,48], an increase in time spent in the center of the open field suggests reductions in anxiety and/or increases in exploration [47]. Diabetic rats also displayed an anxiety-like behavior by reducing exploratory activity in the center zone in the open arena (Figure 5E and F, Z2 path length: *P* < 0.01; Z2 time: *P* < 0.01). Diabetic rats experiencing consecutive R/M hypoglycemia displayed an anxiety-like behavior compared to their non-diabetic counterparts (*post hoc*: Z2 path length: *P* < 0.01; Z2 time: *P* < 0.01).

### R/M hypoglycemia leads to decreased dendritic density and reduced thickness of hippocampal CA1 at delayed time points

**Figure 6:**
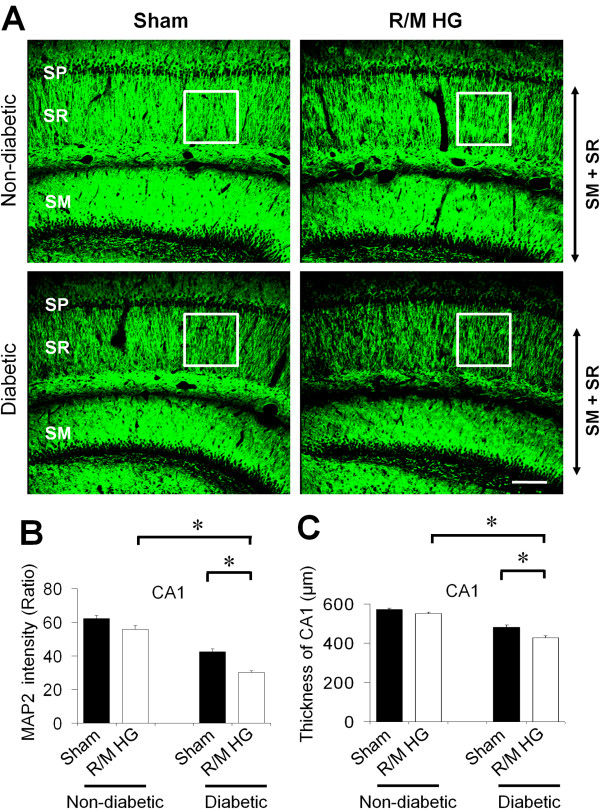
**R/M hypoglycemia decreases dendrite density and thickness of stratum radiatum (SR) and stratum moleculare (SM) layer of hippocampal CA1 8 weeks after injury.** (**A**) Fluorescent images show MAP2 (green) antibody stained stratum radiatum (SR) stratum moleculare (SM) of hippocampal CA1 area 8 weeks after R/M hypoglycemia. The MAP2 intensity in the dendritic area after R/M hypoglycemia (R/M HG) is substantially decreased. This MAP2 intensity decrease is more obvious in the diabetic rats, which suggests R/M hypoglycemia-induced dendrite loss in the SR and SM of hippocampus is worse in the diabetic animal. SP, Stratum Pyramidalae. Bar = 200 μm. (**B**) Bar graph represents quantitated MAP2 fluorescent intensity from the SR area of hippocampal CA1 area. Square in (**A**) shows evaluated area of MAP2 fluorescent intensity. (**C**) Bar graph represents thickness of SR + SM area of hippocampal CA1 area. Data are mean + s.e.m.; *n* = 7, * *P* < 0.05.

To evaluate long-term effects of R/M hypoglycemia on hippocampal dendritic structure, brains were histologically evaluated by MAP2 immunostaining 8 weeks after R/M hypoglycemia (Figure 6A). Here we found that R/M hypoglycemia reduced MAP2 intensity in the SR area of hippocampal CA1. STZ-induced diabetic rats showed significantly (31.65%) less MAP2 immunoreactivity in the SR area of the hippocampus compared with non-diabetic rats. R/M hypoglycemia in diabetic rats further decreases MAP2 intensity (29.03%) than in non-diabetic rats (10.15%) (Figure 6B). The thickness of the CA1 dendritic area was also decreased by 3.81% in non-diabetic rats but by 11.35% in diabetic rats, in concert with the decrease in MAP2 intensity (Figure 6C).

### Inhibition of NADPH oxidase prevents oxidative injury and microglial activation after R/M hypoglycemia

**Figure 7:**
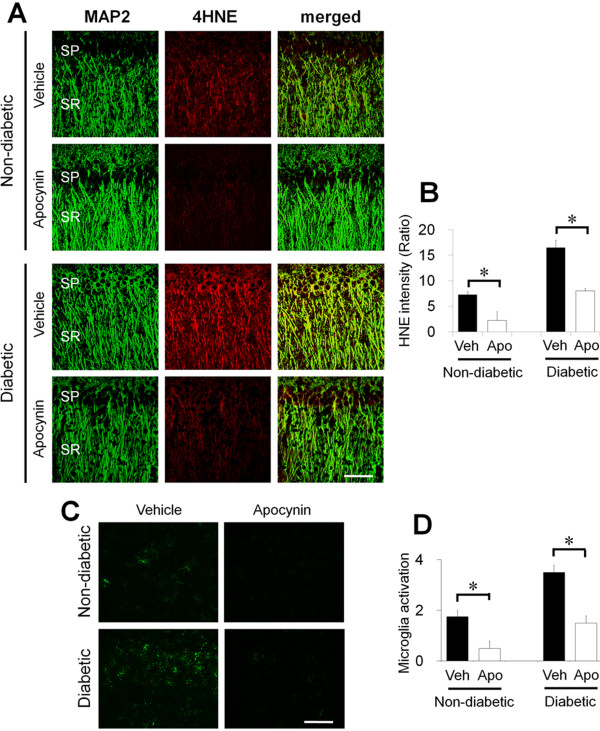
**R/M hypoglycemia-induced dendritic oxidative injury and microglial activation was reduced by the NADPH oxidase inhibitor, apocynin.** (**A**) Fluorescent images show MAP2 (green) and 4HNE (red) antibody stained stratum radiatum (SR) of hippocampal CA1 area. The 4HNE intensity in the dendritic area after R/M hypoglycemia (R/M HG) is substantially increased in diabetic and non-diabetic rats. The NADPH oxidase inhibitor, apocynin (Apo), reduced 4HNE intensity is much higher in the diabetic and in the non-diabetic rats, which suggests R/M hypoglycemia-induced oxidative injury in the dendritic area of hippocampus is mediated by NADPH oxides activation. SP, Stratum pyramidalae. Bar = 100 μm. (**B**) Bar graph represents quantitated 4HNE fluorescent intensity from the SR area of hippocampal CA1 area. Data are mean + s.e.m.; *n* = 7, * *P* < 0.05. (**C**) Brain sections were harvested at 1 day after the last episode of moderate hypoglycemia and immunostained with CD11b. R/M hypoglycemia-induced microglial activation in the hippocampus is apparent in both non-diabetic and diabetic rats. Apocynin (Apo) treatment reduced microglia activation in the diabetic or in the non-diabetic rats. Scale bar = 100 μm. (**D**) Quantification of microglial activation was performed from hippocampal CA1 pyramidal area. Microglial activation is quantified based on morphological change and intensity of CD11b immunostaining. Data are mean + s.e.m.; *n* = 5, * *P* < 0.05.

To test whether NADPH oxidase activation contributes to the dendritic injury seen in the stratum radiatum of hippocampal CA1 area and microglial activation after R/M hypoglycemia, we injected an NADPH oxidase inhibitor, apocynin, into the intraperitoneal space. Apocynin treatment (15 mg/kg/day) reduced 4HNE fluorescence intensity by 51.30% in diabetic rats after hypoglycemia. Reduction of 4HNE fluorescent intensity represents that R/M hypoglycemia-induced oxidative injury may be caused by superoxide production through NADPH oxidase activation. R/M hypoglycemia-induced microglia activation was also reduced by 57.15% by apocynin treatment in diabetic rats. The degree of reduction of 4HNE fluorescent intensity and microglia activation by apocynin was similar in diabetic and non-diabetic rats (Figure 7A and B).

## Discussion

The present study demonstrates that R/M hypoglycemia induces cognitive impairment associated with hippocampal dendritic injury. We found that R/M hypoglycemia induces oxidative injury in hippocampal dendrites and that R/M hypoglycemia induces microglial activation in the hippocampus and cerebral cortex. Both of these processes were more pronounced in diabetic rats. R/M hypoglycemia induces cognitive impairment 6 weeks after insult in diabetic rats, but not in non-diabetic rats. Finally; R/M hypoglycemia reduces dendritic density and thickness of the SR and SM of hippocampal CA1 area.

In the present study we tested if R/M hypoglycemia in diabetic rats could cause hippocampal dendritic injury and cognitive impairment. Severe hypoglycemia (that in which iso- EEG is present) induces neuronal death in the cerebral cortex and hippocampus, and cognitive impairment. However, moderate hypoglycemia produces neuronal death that is restricted to the cerebral cortex [34,49], It is not clear why this variation occurs.

To investigate whether differences in neuronal death depend on a pre-existing diabetic condition before insult, we rendered rats diabetic by STZ injection. The present study showed cortical but not hippocampal neuronal death, similar to what was seen in a previous study [46]. Thus, the pattern of neuronal death in the cerebral cortex or in the hippocampus in the diabetic rats after R/M hypoglycemia is almost identical with other studies, which used non-diabetic rats [34,35,49] .

Whether R/M hypoglycemia causes cognitive impairment remains an important question. Repetitive hypoglycemia in the developing brain causes selective impairment of synaptic plasticity, in the absence of hippocampal neuronal death, suggesting that impaired synaptic plasticity in the hippocampus caused by repetitive hypoglycemia could underlie memory and cognitive deficits observed in diabetic children [35]. In the present study, we tested the hypothesis that R/M hypoglycemia may cause oxidative injury in hippocampal dendrites [16,45]. To do this, we subjected young diabetic and healthy rats to R/M hypoglycemia and evaluated by immunostaining for 4HNE, an α,β-unsaturated hydroxyalkenal produced by lipid peroxidation in cells. 4HNE immunoreactivity in the hippocampal dendritic area was substantially increased after sequential hypoglycemic events, and was higher in diabetic rats compared to non-diabetic rats. Next we asked if oxidative injury could be induced after only a single episode of moderate hypoglycemia. Neither diabetic nor non-diabetic rats showed oxidative injury after only one episode, suggesting that repetitive moderate hypoglycemia events are required to induce oxidative injury in the hippocampal dendritic area. Our results also confirm previous findings that hypoglycemia induces lipid peroxidation in discrete regions of the forebrain [34,50].

Previously, McNay and Sherwin published the intriguing result that R/M hypoglycemia actually led to cognitive improvement in rats [51]. In the study, they performed a spatial working memory test shortly (20 min) after R/M hypoglycemia (3 h daily for 3 days). This improvement was equal in diabetic and non-diabetic rats, in contrast to the present study. However, a major difference between these studies is that behavioral testing in the present study was conducted 6 weeks after R/M hypoglycemia. Another study showed that R/M hypoglycemia prevented subsequent severe hypoglycemia-induced neuronal death and cognitive impairment [38]. This acute preconditioning effect is not relevant to the chronic functional impairment described here.

Microglial activation is also induced by severe hypoglycemia, and can contribute to neuronal injury [52], by releasing several neurotoxic substances, including superoxide, nitric oxide, and metalloproteinase [43,53-56] In the present study, we found that R/M hypoglycemia also induced microglial activation in the hippocampus, as seen in severe hypoglycemia [52], and diabetic rats showed higher microglial activation after R/M hypoglycemia. Thus, this confirms that the inflammatory response was also induced after R/M hypoglycemia, but whether this inflammatory response contributes to the oxidative stress or later cognitive impairment is not established.

Debate continues over the role of hypoglycemia and/or hyperglycemia in producing cognitive impairment in type 1 diabetes. In the present study, non-diabetic rats showed no obvious cognitive impairments after R/M hypoglycemia, but diabetic rats showed statistically significant cognitive impairment. Why was cognitive impairment only seen in the diabetic rats after R/M hypoglycemia? The present study does not provide a clear answer for this; however, we can speculate that less severe oxidative injury in the non-diabetic rats or impaired recovery in the diabetic rats influenced this. In support of these possibilities, we found that R/M hypoglycemia reduced MAP2 intensity and thickness in the stratum radiatum area of hippocampal CA1, more so in diabetic than in non-diabetic rats. Synaptic density in the hippocampus plays a crucial role in memory [57]. For example, synaptic density in the CA1 stratum radiatum is regulated by estrogen, leading to modulation of long-term depression (LTD) and long-term potentiation (LTP) [58]. Evidence suggests that diabetes, stress, and aging negatively affect synaptic plasticity in brain regions including the hippocampus and the cortex, which can lead to persistent inhibition of LTP and facilitation of LTD and in turn might lead to activity-dependent synapse weakening and contribute to cognitive impairments [59].

We found that R/M hypoglycemia-induced oxidative damage in hippocampal dendrites and microglial activation was reduced by the NADPH oxidase inhibitor, apocynin, suggesting a mechanism by which R/M hypoglycemia may promote oxidative stress in the hippocampus during reperfusion. Glucose reperfusion-induced dendritic injury results in part from the activation by superoxide produced through NADPH oxidase. NADPH oxidase generates superoxide through a process that requires glucose as a substrate for NADPH production, such that glucose availability can be the rate-limiting factor in superoxide production by this process [42,60,61].

Extrapolating these results to clinical settings has some limitations. The experimental paradigm of hypoglycemia for 5 consecutive days is intended to model recurrent hypoglycemia, but the effects over time of widely spaced hypoglycemic intervals may differ from the tightly spaced recurrences used here. Similarly, the diabetic rats were subjected to much greater variations in glucose than would typically occur in clinical settings. They also were subjected to much greater changes in plasma glucose than the non-diabetic rats, a factor which may have contributed to the differences observed between the diabetic and non-diabetic rats. Last, rodent brains may differ from human brains in their responses to R/M hypoglycemia. Further research, particularly from the translational and clinical perspective is needed to resolve these questions.

## Abbreviations

ANOVA, Analysis of variance; A/S hypoglycemia, Acute/severe (A/S) hypoglycemia; EEG, Electrocardiogram; CA1, Cornu Ammonis area 1; CD11b, Cluster of differentiation molecule 11b; DMSO, Dimethylsulphoxide; FJB, Fluoro-Jade B; H&E, Haematoxylin/eosin; 4HNE, 4-hydroxy-2-nonenal; i.p, Intraperitoneal; LTD, Long-term depression; LTP, Long term potentiation; MAP2, Microtubule-associated protein 2; NADPH, Nicotinamide-adenine dinucleotide phosphate; RANOVA, Repeated measure of analysis of variance; R/M hypoglycemia, Recurrent/moderate hypoglycemia; SR, Stratum radiatum; STZ, Streptozotocin.

## Competing interests

The authors declare no conflict of interest.

## Author contributions

SW, BY, TK, BC, JK, BJ, ML, and JL researched data. MS performed the data analysis; SW, MS, JL, RS, and SS reviewed and edited the manuscript. RS and SS contributed to the discussion. SS wrote the manuscript. All authors read and approved the final manuscript.

## Supplementary Material

Additional file 1 **Figure S1.** Experimental protocol for R/M hypoglycemia or A/S hypoglycemia in the diabetic rats. Type 1 diabetes rats were induced by 2 consecutive days of intraperitoneal streptozotocin (50 mg/kg) injection. One week after STZ injection, diabetic rats were subjected to either moderate hypoglycemia for 5 consecutive days (**A**) or severe hypoglycemia once (**B**). For moderate hypoglycemia, rats were unanesthetized during entire experiment. One group of rats was sacrificed at 24 h after the last moderate hypoglycemia episode for histological evaluation for neuron death (FJB), oxidative damage (4HNE), and microglial activation (CD11b). The other group of rats was subjected to cognitive function evaluation with the Barnes maze behavioral test 6 weeks after R/M hypoglycemia and then sacrificed for MAP2 staining. For severe hypoglycemia, rats were anesthetized by isoflurane during entire hypoglycemia surgery. Rats were sacrificed 1 week after hypoglycemia for assessment of neuron death (FJB), oxidative damage (4HNE), and microglial activation (CD11b). Click here for file

Additional file 2 **Figure S2. **EEG and blood glucose changes during hypoglycemia in diabetic rats. Insulin injection increases amplitude and reduces frequency of cortical EEG. Iso-EEG (flat line, e) was induced 4 h after 30 U/kg insulin injections. Initial fasting blood glucose concentration from diabetic rats in this experiment was above 15 mM. This blood glucose concentration was quickly decreased immediately after insulin injection. During the iso-EEG, blood glucose concentration was below 0.5 mM. After glucose reperfusion (G/R), blood glucose concentration was increased above 9 mM. Click here for file

## References

[B1] Sommerfield AJ Deary IJ McAulay V Frier BM Short-term, delayed, and working memory are impaired during hypoglycemia in individuals with type 1 diabetes Diabetes Care 2003 26 390 396 10.2337/diacare.26.2.390 12547868

[B2] Zammitt NN Streftaris G Gibson GJ Deary IJ Frier BM Modeling the consistency of hypoglycemic symptoms: high variability in diabetes Diabetes Technol Ther 2011 13 571 578 10.1089/dia.2010.0207 21413888

[B3] Blasetti A Chiuri RM Tocco AM Di Giulio C Mattei PA Ballone E Chiarelli F Verrotti A The effect of recurrent severe hypoglycemia on cognitive performance in children with type 1 diabetes: a meta-analysis J Child Neurol 2011 26 1383 1391 10.1177/0883073811406730 21572053

[B4] Frier BM Cognitive functioning in type 1 diabetes: the Diabetes Control and Complications Trial (DCCT) revisited Diabetologia 2011 54 233 236 10.1007/s00125-010-1983-6 21109995

[B5] Golden MP Ingersoll GM Brack CJ Russell BA Wright JC Huberty TJ Longitudinal relationship of asymptomatic hypoglycemia to cognitive function in IDDM Diabetes Care 1989 12 89 93 10.2337/diacare.12.2.89 2702906

[B6] Ryan CM Atchison J Puczynski S Puczynski M Arslanian S Becker D Mild hypoglycemia associated with deterioration of mental efficiency in children with insulin-dependent diabetes mellitus J Pediatr 1990 117 32 38 10.1016/S0022-3476(05)82440-0 2196358

[B7] Warren RE Frier BM Hypoglycaemia and cognitive function Diabetes Obes Metab 2005 7 493 503 10.1111/j.1463-1326.2004.00421.x 16050942

[B8] Wright RJ Frier BM Deary IJ Effects of acute insulin-induced hypoglycemia on spatial abilities in adults with type 1 diabetes Diabetes Care 2009 32 1503 1506 10.2337/dc09-0212 19487633 PMC2713616

[B9] Auer RN Hypoglycemic brain damage Forensic Sci Int 2004 146 105 110 10.1016/j.forsciint.2004.08.001 15542270

[B10] Davis EA Jones TW Hypoglycemia in children with diabetes: incidence, counterregulation and cognitive dysfunction J Pediatr Endocrinol Metab 1998 Suppl 1 177 182 9642657 10.1515/jpem.1998.11.s1.177

[B11] Lincoln NB Faleiro RM Kelly C Kirk BA Jeffcoate WJ Effect of long-term glycemic control on cognitive function Diabetes Care 1996 19 656 658 10.2337/diacare.19.6.656 8725868

[B12] Sucov A Woolard RH Ethanol-associated hypoglycemia is uncommon Acad Emerg Med 1995 2 185 189 10.1111/j.1553-2712.1995.tb03192.x 7497031

[B13] Auer RN Olsson Y Siesjo BK Hypoglycemic brain injury in the rat. Correlation of density of brain damage with the EEG isoelectric time: a quantitative study Diabetes 1984 33 1090 1098 10.2337/diabetes.33.11.1090 6500189

[B14] Auer RN Wieloch T Olsson Y Siesjo BK The distribution of hypoglycemic brain damage Acta Neuropathol 1984 64 177 191 10.1007/BF00688108 6496035

[B15] Suh SW Aoyama K Chen Y Garnier P Matsumori Y Gum E Liu J Swanson RA Hypoglycemic neuronal death and cognitive impairment are prevented by poly (ADP-ribose) polymerase inhibitors administered after hypoglycemia J Neurosci 2003 23 10681 10690 14627653 10.1523/JNEUROSCI.23-33-10681.2003 PMC6740913

[B16] Auer RN Kalimo H Olsson Y Siesjo BK The temporal evolution of hypoglycemic brain damage. I. Light- and electron-microscopic findings in the rat cerebral cortex Acta Neuropathol 1985 67 13 24 10.1007/BF00688120 4024866

[B17] Kalimo H Olsson Y Effects of severe hypoglycemia on the human brain Neuropathological case reports. Acta Neurol Scand 1980 62 345 356 10.1111/j.1600-0404.1980.tb03047.x 6781212

[B18] Malouf R Brust JC Hypoglycemia: causes, neurological manifestations, and outcome Ann Neurol 1985 17 421 430 10.1002/ana.410170502 4004166

[B19] Auer RN Hugh J Cosgrove E Curry B Neuropathologic findings in three cases of profound hypoglycemia Clin Neuropathol 1989 8 63 68 2721042

[B20] Langan SJ Deary IJ Hepburn DA Frier BM Cumulative cognitive impairment following recurrent severe hypoglycaemia in adult patients with insulin-treated diabetes mellitus Diabetologia 1991 34 337 344 10.1007/BF00405006 1864488

[B21] Ben-Ami H Nagachandran P Mendelson A Edoute Y Drug-induced hypoglycemic coma in 102 diabetic patients Arch Intern Med 1999 159 281 284 10.1001/archinte.159.3.281 9989540

[B22] Ennis K Tran PV Seaquist ER Rao R Postnatal age influences hypoglycemia-induced neuronal injury in the rat brain Brain Res 2008 1224 119 126 18582442 10.1016/j.brainres.2008.06.003 PMC2584018

[B23] Brands AM Biessels GJ de Haan EH Kappelle LJ Kessels RP The effects of type 1 diabetes on cognitive performance: a meta-analysis Diabetes Care 2005 28 726 735 10.2337/diacare.28.3.726 15735218

[B24] Hershey T Perantie DC Warren SL Zimmerman EC Sadler M White NH Frequency and timing of severe hypoglycemia affects spatial memory in children with type 1 diabetes Diabetes Care 2005 28 2372 2377 10.2337/diacare.28.10.2372 16186265

[B25] Deary IJ Crawford JR Hepburn DA Langan SJ Blackmore LM Frier BM Severe hypoglycemia and intelligence in adult patients with insulin-treated diabetes Diabetes 1993 42 341 344 10.2337/diabetes.42.2.341 8425671

[B26] Rovet J Alvarez M Attentional functioning in children and adolescents with IDDM Diabetes Care 1997 20 803 810 10.2337/diacare.20.5.803 9135946

[B27] Bjorgaas M Gimse R Vik T Sand T Cognitive function in type 1 diabetic children with and without episodes of severe hypoglycaemia Acta Paediatr 1997 86 148 153 10.1111/j.1651-2227.1997.tb08856.x 9055883

[B28] Perantie DC Lim A Wu J Weaver P Warren SL Sadler M White NH Hershey T Effects of prior hypoglycemia and hyperglycemia on cognition in children with type 1 diabetes mellitus Pediatr Diabetes 2008 9 87 95 10.1111/j.1399-5448.2007.00274.x 18208449

[B29] Gerich JE Mokan M Veneman T Korytkowski M Mitrakou A Hypoglycemia unawareness Endocr Rev 1991 12 356 371 10.1210/edrv-12-4-356 1760993

[B30] Cryer PE Hypoglycemia-associated autonomic failure in diabetes Am J Physiol Endocrinol Metab 2001 281 E1115 E1121 11701423 10.1152/ajpendo.2001.281.6.E1115

[B31] Zammitt NN Warren RE Deary IJ Frier BM Delayed recovery of cognitive function following hypoglycemia in adults with type 1 diabetes: effect of impaired awareness of hypoglycemia Diabetes 2008 57 732 736 10.2337/db07-0695 18039813

[B32] Tallroth G Lindgren M Stenberg G Rosen I Agardh CD Neurophysiological changes during insulin-induced hypoglycaemia and in the recovery period following glucose infusion in type 1 (insulin-dependent) diabetes mellitus and in normal man Diabetologia 1990 33 319 323 10.1007/BF00403327 2198189

[B33] McCrimmon RJ Frier BM Hypoglycaemia, the most feared complication of insulin therapy Diabete Metab 1994 20 503 512 7713272

[B34] Haces ML Montiel T Massieu L Selective vulnerability of brain regions to oxidative stress in a non-coma model of insulin-induced hypoglycemia Neuroscience 2010 165 28 38 10.1016/j.neuroscience.2009.10.003 19818385

[B35] Yamada KA Rensing N Izumi Y De Erausquin GA Gazit V Dorsey DA Herrera DG Repetitive hypoglycemia in young rats impairs hippocampal long-term potentiation Pediatr Res 2004 55 372 379 10.1203/01.PDR.0000110523.07240.C1 14681492

[B36] Kilkenny C Improving bioscience research reporting: the ARRIVE guidelines for reporting animal research PLoS Biol 2012 8 e1000412 20613859 10.1371/journal.pbio.1000412 PMC2893951

[B37] Deeds MC Anderson JM Armstrong AS Gastineau DA Hiddinga HJ Jahangir A Eberhardt NL Kudva YC Single dose streptozotocin-induced diabetes: considerations for study design in islet transplantation models Lab Anim 2011 45 131 140 10.1258/la.2010.010090 21478271 PMC3917305

[B38] Puente EC Silverstein J Bree AJ Musikantow DR Wozniak DF Maloney S Daphna-Iken D Fisher SJ Recurrent moderate hypoglycemia ameliorates brain damage and cognitive dysfunction induced by severe hypoglycemia Diabetes 2010 59 1055 1062 10.2337/db09-1495 20086229 PMC2844814

[B39] Peters EA Hiltermann JT Stolk J Effect of apocynin on ozone-induced airway hyperresponsiveness to methacholine in asthmatics Free Radic Biol Med 2001 31 1442 1447 10.1016/S0891-5849(01)00725-0 11728816

[B40] Suh SW Chen JW Motamedi M Bell B Listiak K Pons NF Danscher G Frederickson CJ Evidence that synaptically-released zinc contributes to neuronal injury after traumatic brain injury Brain Res 2000 852 268 273 10.1016/S0006-8993(99)02095-8 10678752

[B41] Schmued LC Hopkins KJ Fluoro-Jade: novel fluorochromes for detecting toxicant-induced neuronal degeneration Toxicol Pathol 2000 28 91 99 10.1177/019262330002800111 10668994

[B42] Suh SW Gum ET Hamby AM Chan PH Swanson RA Hypoglycemic neuronal death is triggered by glucose reperfusion and activation of neuronal NADPH oxidase J Clin Invest 2007 117 910 918 10.1172/JCI30077 17404617 PMC1838937

[B43] Kauppinen TM Higashi Y Suh SW Escartin C Nagasawa K Swanson RA Zinc triggers microglial activation J Neurosci 2008 28 5827 5835 10.1523/JNEUROSCI.1236-08.2008 18509044 PMC2680357

[B44] Barnes CA Memory deficits associated with senescence: a neurophysiological and behavioral study in the rat J Comp Physiol Psychol 1979 93 74 104 221551 10.1037/h0077579

[B45] Auer RN Siesjo BK Hypoglycaemia: brain neurochemistry and neuropathology Baillieres Clin Endocrinol Metab 1993 7 611 625 10.1016/S0950-351X(05)80210-1 8379907

[B46] Bree AJ Puente EC Daphna-Iken D Fisher SJ Diabetes increases brain damage caused by severe hypoglycemia Am J Physiol Endocrinol Metab 2009 297 E194 E201 10.1152/ajpendo.91041.2008 19435850 PMC2711670

[B47] Dulawa SC Grandy DK Low MJ Paulus MP Geyer MA Dopamine D4 receptor-knock-out mice exhibit reduced exploration of novel stimuli J Neurosci 1999 19 9550 9556 10531457 10.1523/JNEUROSCI.19-21-09550.1999 PMC6782928

[B48] Prut L Belzung C The open field as a paradigm to measure the effects of drugs on anxiety-like behaviors: a review Eur J Pharmacol 2003 463 3 33 10.1016/S0014-2999(03)01272-X 12600700

[B49] Tkacs NC Pan Y Raghupathi R Dunn-Meynell AA Levin BE Cortical Fluoro-Jade staining and blunted adrenomedullary response to hypoglycemia after noncoma hypoglycemia in rats J Cereb Blood Flow Metab 2005 25 1645 1655 10.1038/sj.jcbfm.9600152 15902194

[B50] Patockova J Marhol P Tumova E Krsiak M Rokyta R Stipek S Crkovska J Andel M Oxidative stress in the brain tissue of laboratory mice with acute post insulin hypoglycemia Physiol Res 2003 52 131 135 12625818

[B51] McNay EC Sherwin RS Effect of recurrent hypoglycemia on spatial cognition and cognitive metabolism in normal and diabetic rats Diabetes 2004 53 418 425 10.2337/diabetes.53.2.418 14747293

[B52] Shin BS Won SJ Yoo BH Kauppinen TM Suh SW Prevention of hypoglycemia-induced neuronal death by hypothermia J Cereb Blood Flow Metab 2010 30 390 402 10.1038/jcbfm.2009.229 19861976 PMC2949122

[B53] Chao CC Hu S Molitor TW Shaskan EG Peterson PK Activated microglia mediate neuronal cell injury via a nitric oxide mechanism J Immunol 1992 149 2736 2741 1383325

[B54] Giulian D Vaca K Corpuz M Brain glia release factors with opposing actions upon neuronal survival J Neurosci 1993 13 29 37 8423475 10.1523/JNEUROSCI.13-01-00029.1993 PMC6576318

[B55] Hanisch UK Microglia as a source and target of cytokines Glia 2002 40 140 155 10.1002/glia.10161 12379902

[B56] Vilhardt F Microglia: phagocyte and glia cell Int J Biochem Cell Biol 2005 37 17 21 10.1016/j.biocel.2004.06.010 15381143

[B57] Hara Y Park CS Janssen WG Punsoni M Rapp PR Morrison JH Synaptic characteristics of dentate gyrus axonal boutons and their relationships with aging, menopause, and memory in female rhesus monkeys J Neurosci 2011 31 7737 7744 10.1523/JNEUROSCI.0822-11.2011 21613486 PMC3103072

[B58] Foy MR 17beta-estradiol: effect on CA1 hippocampal synaptic plasticity Neurobiol Learn Mem 2001 76 239 252 10.1006/nlme.2001.4018 11726235

[B59] Artola A Diabetes-, stress- and ageing-related changes in synaptic plasticity in hippocampus and neocortex-the same metaplastic process? Eur J Pharmacol 2008 585 153 162 10.1016/j.ejphar.2007.11.084 18395200

[B60] Decoursey TE Ligeti E Regulation and termination of NADPH oxidase activity Cell Mol Life Sci 2005 62 2173 2193 10.1007/s00018-005-5177-1 16132232 PMC11139152

[B61] Suh SW Shin BS Ma H Van Hoecke M Brennan AM Yenari MA Swanson RA Glucose and NADPH oxidase drive neuronal superoxide formation in stroke Ann Neurol 2008 64 654 663 10.1002/ana.21511 19107988 PMC4304737

