# Supplementary figures and images for: Recurrent/moderate hypoglycemia induces hippocampal dendritic injury, microglial activation, and cognitive impairment in diabetic rats
Source: J Neuroinflammation. 2012 Jul 25;9:182. doi: 10.1186/1742-2094-9-182 (PMC3458941; doi:10.1186/1742-2094-9-182)

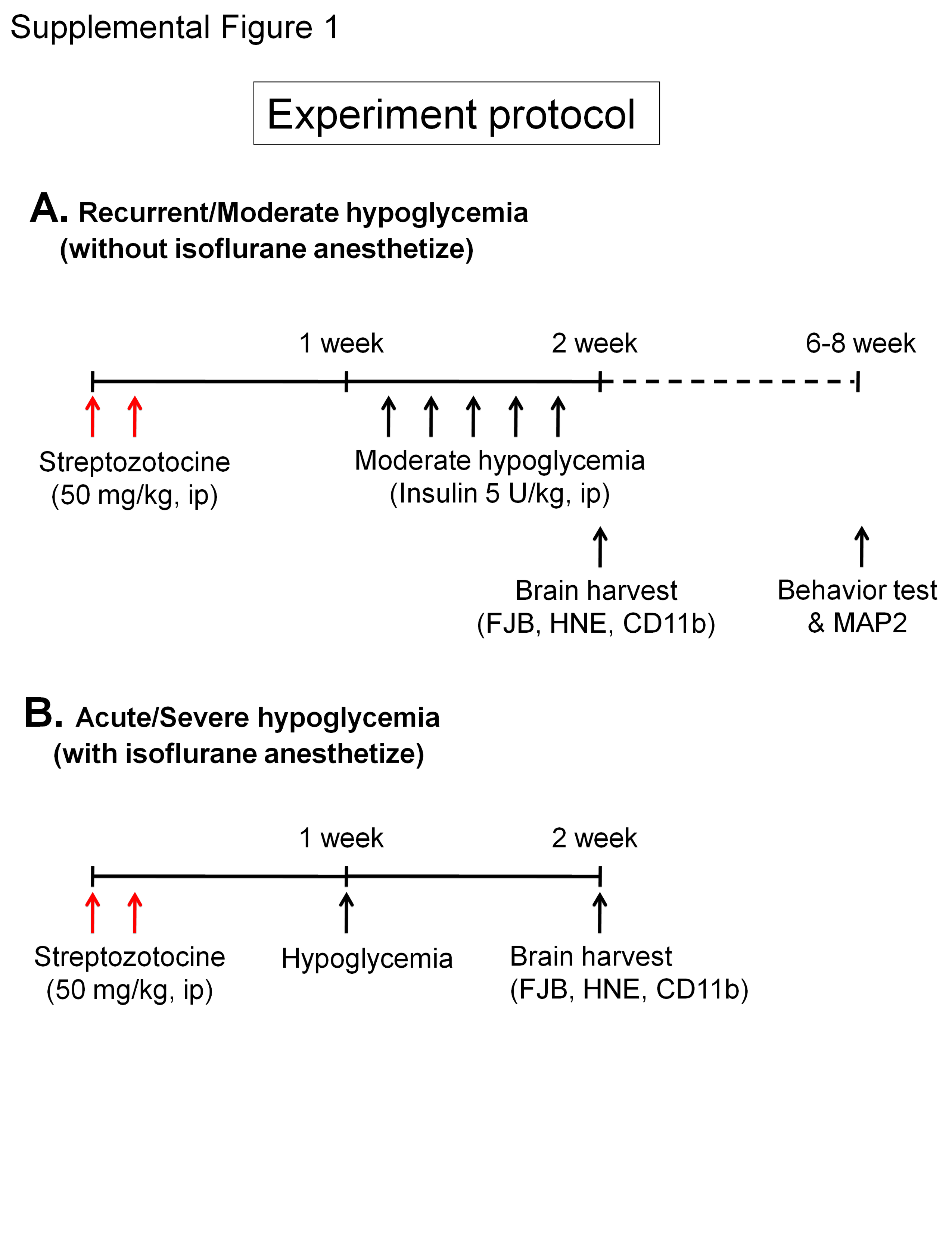

Supplement: Additional file 1 — Figure S1. Experimental protocol for R/M hypoglycemia or A/S hypoglycemia in the diabetic rats. Type 1 diabetes rats were induced by 2 consecutive days of intraperitoneal streptozotocin (50 mg/kg) injection. One week after STZ injection, diabetic rats were subjected to either moderate hypoglycemia for 5 consecutive days (A) or severe hypoglycemia once (B). For moderate hypoglycemia, rats were unanesthetized during entire experiment. One group of rats was sacrificed at 24 h after the last moderate hypoglycemia episode for histological evaluation for neuron death (FJB), oxidative damage (4HNE), and microglial activation (CD11b). The other group of rats was subjected to cognitive function evaluation with the Barnes maze behavioral test 6 weeks after R/M hypoglycemia and then sacrificed for MAP2 staining. For severe hypoglycemia, rats were anesthetized by isoflurane during entire hypoglycemia surgery. Rats were sacrificed 1 week after hypoglycemia for assessment of neuron death (FJB), oxidative damage (4HNE), and microglial activation (CD11b). [file 1742-2094-9-182-S1.tiff]

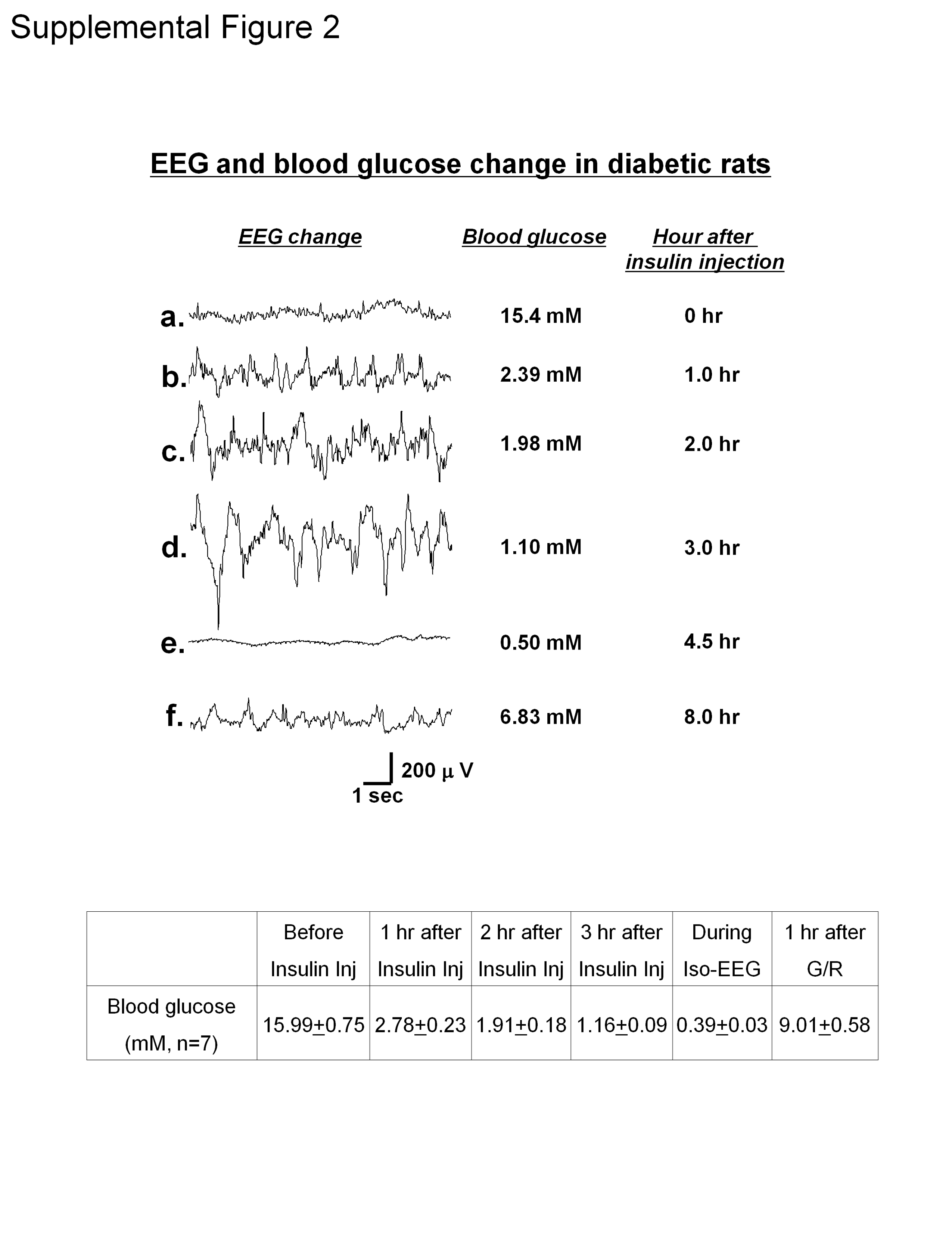

Supplement: Additional file 2 — Figure S2. EEG and blood glucose changes during hypoglycemia in diabetic rats. Insulin injection increases amplitude and reduces frequency of cortical EEG. Iso-EEG (flat line, e) was induced 4 h after 30 U/kg insulin injections. Initial fasting blood glucose concentration from diabetic rats in this experiment was above 15 mM. This blood glucose concentration was quickly decreased immediately after insulin injection. During the iso-EEG, blood glucose concentration was below 0.5 mM. After glucose reperfusion (G/R), blood glucose concentration was increased above 9 mM. [file 1742-2094-9-182-S2.tiff]
